# R on the side of caution

**DOI:** 10.1007/s12471-021-01629-9

**Published:** 2021-09-22

**Authors:** L. Baris, E. J. van den Bos

**Affiliations:** Albert Schweitzer Hospital, Dordrecht, The Netherlands

A 52-year-old man known to suffer from dilated cardiomyopathy due to a mutation of the *LMNA* gene was admitted to the cardiac intensive care unit in cardiogenic shock. He had a cardiac resynchronisation device (CRT-D) programmed at a paced atrioventricular delay of 200 ms and a lower rate set at 80 beats/min. The electrocardiogram at admission is shown in Fig. 1.

**Fig. 1:**
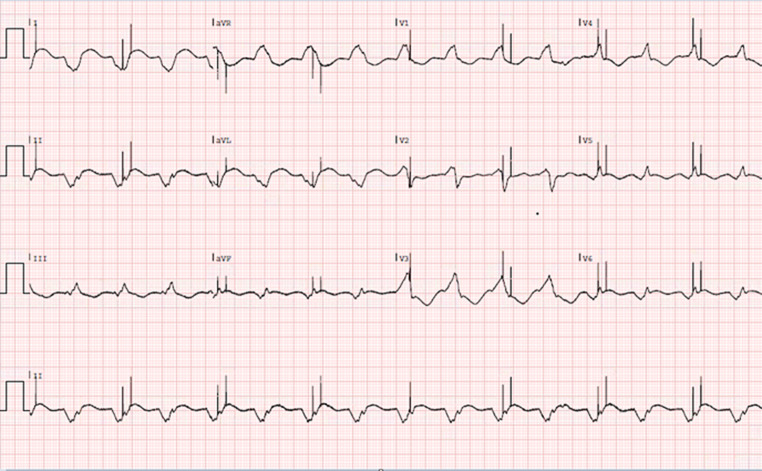
Electrocardiogram at presentation

What rhythm is seen and how can the pacing activity be explained?

## Answer

You will find the answer elsewhere in this issue.

